# ESSENTIALS: Software for Rapid Analysis of High Throughput Transposon Insertion Sequencing Data

**DOI:** 10.1371/journal.pone.0043012

**Published:** 2012-08-10

**Authors:** Aldert Zomer, Peter Burghout, Hester J. Bootsma, Peter W. M. Hermans, Sacha A. F. T. van Hijum

**Affiliations:** 1 Laboratory of Pediatric Infectious Diseases, Radboud University Medical Centre, Nijmegen, The Netherlands; 2 Centre for Molecular and Biomolecular Informatics, Nijmegen Centre for Molecular Life Sciences, Radboud University Medical Centre, Nijmegen, The Netherlands; 3 NIZO Food Research, Kluyver Centre for Genomics of Industrial Fermentation, Ede, The Netherlands; 4 TI Food and Nutrition, Wageningen, The Netherlands; 5 Netherlands Bioinformatics Centre, Nijmegen, The Netherlands; University of Toronto, Canada

## Abstract

High-throughput analysis of genome-wide random transposon mutant libraries is a powerful tool for (conditional) essential gene discovery. Recently, several next-generation sequencing approaches, e.g. Tn-seq/INseq, HITS and TraDIS, have been developed that accurately map the site of transposon insertions by mutant-specific amplification and sequence readout of DNA flanking the transposon insertions site, assigning a measure of essentiality based on the number of reads per insertion site flanking sequence or per gene. However, analysis of these large and complex datasets is hampered by the lack of an easy to use and automated tool for transposon insertion sequencing data. To fill this gap, we developed ESSENTIALS, an open source, web-based software tool for researchers in the genomics field utilizing transposon insertion sequencing analysis. It accurately predicts (conditionally) essential genes and offers the flexibility of using different sample normalization methods, genomic location bias correction, data preprocessing steps, appropriate statistical tests and various visualizations to examine the results, while requiring only a minimum of input and hands-on work from the researcher. We successfully applied ESSENTIALS to in-house and published Tn-seq, TraDIS and HITS datasets and we show that the various pre- and post-processing steps on the sequence reads and count data with ESSENTIALS considerably improve the sensitivity and specificity of predicted gene essentiality.

## Introduction

Discovery of microbial genes essential for growth, survival and/or pathogenesis has frequently been used for gene functional analysis, determining the minimal functional genome and identification of therapeutic targets [1]–[3]. Traditionally, this approach involves extensive testing of fitness defects of single mutants during relevant *in vitro* or *in vivo* conditions with methods that are far from high-throughput.

A first step towards high-throughput screening for essential genes was made with Signature Tagged Mutagenesis (STM) [4]. STM screens single mutants in pools of up to 96 tagged mutants, and readout of mutant pools before and after conditional challenge occurs by Southern hybridization detection of mutant-specific DNA tags. Microarray-based methods for detection of transposon mutants, e.g. Transposon Site Hybridization (TraSH) and Genomic Array Footprinting (GAF) [5]–[7], further improved the throughput of microbial essential gene discovery. However, these approaches potentially suffer from cross-hybridization and lack of resolution hampering the identification of the exact location of the transposon insertion site [8], [9]. These problems were alleviated by high-throughput transposon insertion sequencing analysis methods such as Tn-seq or TraDIS or variants thereof [10]–[13].

With transposon sequencing analysis the presence of each unique mutant within a defined or random transposon mutant library is determined by amplification of DNA flanking the transposon insertion site followed by massively parallel sequencing. Sequence reads from DNA flanking transposon insertion sites are mapped on the reference genome and summarized for each insertion and gene, generating a measurement of fitness for every knockout in comparison to the expected values based on mutant library size, number of possible unique insertion sites per gene and number of sequence reads. Similarly, to identify conditionally essential genes, these libraries are exposed to a challenge condition that will induce loss of mutants of genes essential for survival in these conditions. A measurement of fitness for every knockout comparison between challenge and control condition can then be calculated. In summary, with this technique (i) the decreased fitness of mutants can be detected, (ii) the ubiquity of a specific mutant can be counted and compared to all other mutants in the mutant library and (iii) the exact location of the transposon insertion can be determined.

This method has been successfully applied to determine gene essentiality and the minimal genome of *Streptococcus pneumoniae* TIGR4, *Salmonella typhi* TY2, *Mycobacterium tuberculosis*, *Caulobacter crescentus* and others, and has also been used to pinpoint genes necessary for survival under challenging conditions, such as during colonization or when exposed to antimicrobial chemicals [10], [14]–[18].

Unfortunately, analysis of these next-gen sequencing datasets is hampered by the lack of an easy-to-use and automated method to process the gigabytes of sequence data generated by these methods. Automated download and processing of read files, filtering of spurious ‘contaminating’ reads on presence of transposon sequence and handling ‘barcode’ sequences which uniquely assign reads to a specific sample is still a laborious and hands-on task, only suited for bioinformaticians experienced in next-gen sequencing analysis. Furthermore, various steps have to be taken to improve data quality of transposon insertion sequencing analysis, such as filtering of input data for repetitive sequences, removal of sequence reads from transposons inserted in the 3′ end of a gene that do not cause loss of function, correcting read count for insertion biases introduced by the presence of multiple replication forks in bacteria [19] and proper normalization methods and statistics suitable for next-gen sequencing count data [20], [21].

We developed ESSENTIALS, an open source, web-based software tool suitable for researchers in the genomics field utilizing transposon insertion sequencing analysis. It accurately predicts (conditionally) essential genes and offers the flexibility of using different sample normalization methods, genomic location bias correction, data preprocessing steps, appropriate statistical tests and various visualizations to examine the results. Additionally we show that the various pre- and post-processing steps of the sequence reads and count data with ESSENTIALS considerably improves both sensitivity and specificity of predicted gene essentiality.

## Results and Discussion

To facilitate the analysis of data generated by transposon insertion sequencing by genomics researchers we developed a web-based tool that downloads and processes read files provided by a sequencing facility, filters contaminating reads and splits multi-sample read files on ‘barcode’ sequences that uniquely assign reads to a specific sample. These reads are then mapped onto the relevant genome using pass [22], while non-informative reads, such as reads from repetitive sequences or reads from transposons inserted in the 3′ end of a gene that do not cause loss of function, are removed. Count data per transposon insertion and per gene is calculated and corrected for biases introduced by the presence of multiple replication forks in bacteria [23]. Finally, normalization on replicate samples and statistics suitable for next-gen sequencing count data [20], [24] is applied and presented to the user as tables, figures and an interactive genomic view using MINOMICS [25].

### Benchmarking Datasets

To determine the performance and the optimal processing parameters of ESSENTIALS, we performed a Tn-seq experiment on *S. pneumoniae* R6 because it is one of the most well researched organisms regarding gene essentiality. The experiment was performed in duplicate on two *S. pneumoniae* R6 mutant libraries of approximately 40,000 and 15,000 colony forming units (CFU) respectively (see text S1 for a detailed description of the materials and methods used). Fold change under-representation and statistics of 49 known essential and 49 known non-essential genes of *S. pneumoniae* R6, obtained from OGEEDB [26], were generated using various settings of the ESSENTIALS tool and analyzed with (i) Receiver Operator Characteristics (ROC) on the fold changes to determine if the various steps decreased the number of false positives and false negatives and with (ii) a T-test to examine if the fold changes of essential genes and non-essential genes were significantly different. Input data for ROC analysis and T-testing is given in table S1, including literature references. Genes were considered essential if the associated FDR adjusted p-value of the experiment replicates was <0.05 and if the ratio of the expected number of reads, calculated from the number of possible insertion sites per gene, was lower than the fold change cut-off predicted by ESSENTIALS. A detailed description about the functions of all *S. pneumoniae* R6 essential genes found will be given elsewhere [27].

To benchmark detection of conditionally essential genes in which Tn-seq libraries are compared, we obtained Illumina sequence reads used for determining genes involved in tobramycin resistance in *P. aeruginosa* PAO1 [10] from the EBI Short Read Archive [28]. The effect of gene knockouts of *P. aeruginosa* PAO1 on the minimum inhibitory concentration (MIC) of tobramycin has been comprehensively analyzed [10], [29], and 12 mutants without a tobramycin phenotype and 31 mutants with a 4 fold lower tobramycin MIC are described in these studies. Fold change under-representation and statistics of the 31 known essential and 12 known non-essential genes for *P. aeruginosa* PAO1 tobramycin resistance were generated using various settings of the ESSENTIALS tool and analyzed with Receiver Operator Characteristics (ROC) and T-testing as described above for *S. pneumoniae* (table S2). A gene was considered essential when it had a 2-fold lower number of read counts per gene in the tobramycin stressed condition compared to the reference condition and an associated FDR adjusted p-value <0.05 of the experimental replicates. In the analysis performed by Gallagher, [10], this fold change cut-off resulted in 117 genes predicted to be required for tobramycin resistance.

### Removal of Reads Mapping in Repeat Regions or in the 3′ Terminus Improves Essential Gene Detection

Insertion sites that do not have unique flanking sequences cannot be assigned to a single gene and are as such not informative for gene essentiality. For instance in *S. pneumoniae* R6 more than 5% of the insertion site flanking sequences have a perfect match elsewhere on the genome (results not shown). Failure to remove these reads will result in assigning reads to essential genes, causing these potentially to be detected as non-essential. Reads with a perfect match elsewhere on the genome sequence were excluded, which resulted in removal of 12,006 insertion site flanking sequences, which in turn resulted in an additional 17 essential genes detected for *S. pneumoniae* R6. ROC analysis of essential versus non-essential genes showed an increased area under the curve (AUC) (Table 1). For *P. aeruginosa PAO1*, after repeat-filtering, five genes were no longer considered essential for tobramycin resistance. Repeat filtering did not result in an increase in predictive power of essential genes, as the AUC did not increase (Table 1).

**Table 1:** Effect of statistical methods on the prediction of essential genes based on two datasets.

Experiment	Applied processing step	Essential genes detected*	Predictive value		
			AUC	std.error	P
Essential *S. pneumoniae* R6	**Scaling**	288	0.9517	2.23E-02	1.11E-22
	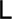 **Repeatfiltering**	305	0.9588	1.83E-02	6.36E-23
	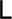 **Gene truncation**	358	0.9996	7.12E-04	3.20E-38
	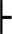 Quantile normalization	359	0.9996	7.12E-04	6.08E-38
	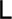 **TMM normalization**	342	1.0000	0.00E+00	1.26E-37
	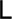 **Genomic location bias correction**	339	1.0000	0.00E+00	3.36E-40
Essential for tobramycinresistance *P. aeruginosa* PAO1	**Scaling**	185	0.6774	8.06E-02	1.27E-02
	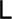 **Repeatfiltering**	180	0.6747	8.07E-02	1.29E-02
	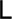 **Gene truncation**	173	0.6667	8.06E-02	1.08E-02
	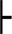 Quantile normalization	174	0.6640	8.10E-02	1.06E-02
	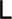 **TMM normalization**	190	0.6640	8.10E-02	1.09E-02
	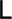 **Genomic location bias correction**	121	0.7634	7.40E-02	2.16E-03
	Literature	117	0.7406	7.56E-02	2.66E-03

The predictive value of each method was assessed using ROC curves and a Welch T-test.

* Cut-offs for *S. pneumoniae* R6 were automatically detected by ESSENTIALS while for *P. aeruginosa PAO1* a cut-off of 2.5 fold underrepresentation of reads per gene in the challenge condition was used to facilitate comparison with the literature data from Gallagher *et al.*

**Figure 1:**
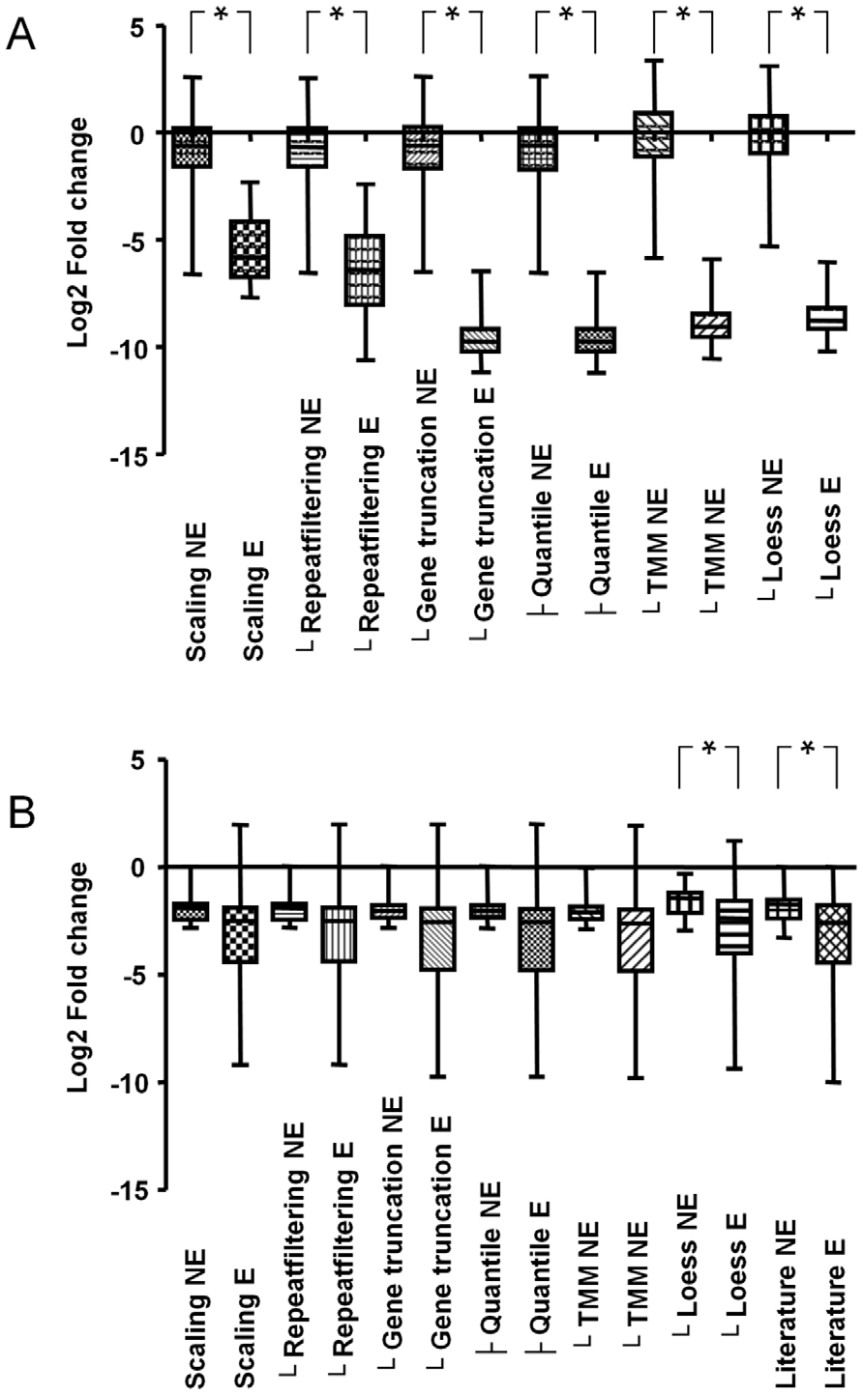
Box whisker plots of gene essentiality data. Box whisker plot showing the sample minimum, lower quartile, median, upper quartile, and sample maximum of (A) fold change data of essential (E) and nonessential (NE) genes for growth of *S. pneumoniae* and (B) fold change data of essential (E) and nonessential (NE) genes for tobramycin resistance of *P. aeruginosa* PAO1 as calculated by ESSENTIALS after the various processing steps and in the case of *P. aeruginosa* PAO1 also for the fold change data presented by Gallagher *et al.* [10]. Significant difference between the essential and non-essential gene distributions is shown by *(p<0.01).

Likewise, transposons that are inserted in the 3′ terminus of a gene often might not lead to loss of function of that gene. Removal of reads mapped to these insertions resulted in an additional 53 essential genes in *S. pneumoniae* R6, a near-perfect AUC and consequently a dramatic reduction of the P-value (Table 1; Fig. 1A). For *P. aeruginosa* PAO1 a small decrease in the AUC but a minor improvement of p-value was observed, however the two populations of essential genes and non-essential genes still could not be considered significantly different (p<0.01) (Table 1; Fig. 1B).

### Normalization Improves Detection of Essential Genes

In many published transposon insertion sequencing analysis studies, information regarding experimental variability from the replicate experiments is not used in statistical testing for gene essentiality. For instance Langridge and co-workers combined the reads per gene of replicate target and control experiments, added an arbitrary value of 100 to all read counts, and calculated ratio’s followed by testing for deviation from a normal distribution [30]. To determine the experimental variability, replicate experiments of the same mutant library should be performed and compared using appropriate statistical tests. Sequencing a sample to half the read-depth compared to other samples will yield half the number of reads mapping to each gene. To facilitate comparisons between samples of varying read-depth, scaling of the samples to their total number of reads is therefore required. Simple total read count scaling is not always appropriate; the number of reads assigned to a gene is not only dependent on the essentiality and the number of insertion sites, but also on the composition of the mutant library after treatment. If many mutants survive in one experimental condition but not in the other, read counts for the remaining genes in that sample are increased. Additionally, PCR artifacts can cause strong over-representation of a single mutant. These artifacts can cause a skew, resulting in decreased specificity and sensitivity, similarly to what is described for RNA-seq data [20].

In microarray data analysis, Quantile normalization [31] is frequently used to correct compositional bias by making distributions of microarray data identical in statistical properties. Use of Quantile normalization on Tn-seq data had a negligent effect on data quality, and prediction of essential genes for *S. pneumoniae* was still not 100% correct as the AUC had not reached 1 yet (Table 1). Recently, a method was proposed by Robinson *et al.* [20] for normalizing next-gen sequencing count data, named the trimmed mean of M values (TMM). TMM and its closely related method Relative Log Expression (RLE) trim an upper and lower fraction of the data and use the remaining data to calculate normalization factors. Application of the TMM normalization method to the *S. pneumoniae* R6 datasets resulted in better separation of essential and non-essential genes judged from the increase in AUC values (Table 1; Fig. 1A). Application of the RLE method gave near-identical results compared to TMM normalization (results not shown).

### Genomic Location Insertion and Read Count Bias can be Corrected by LOESS Regression

**Figure 2:**
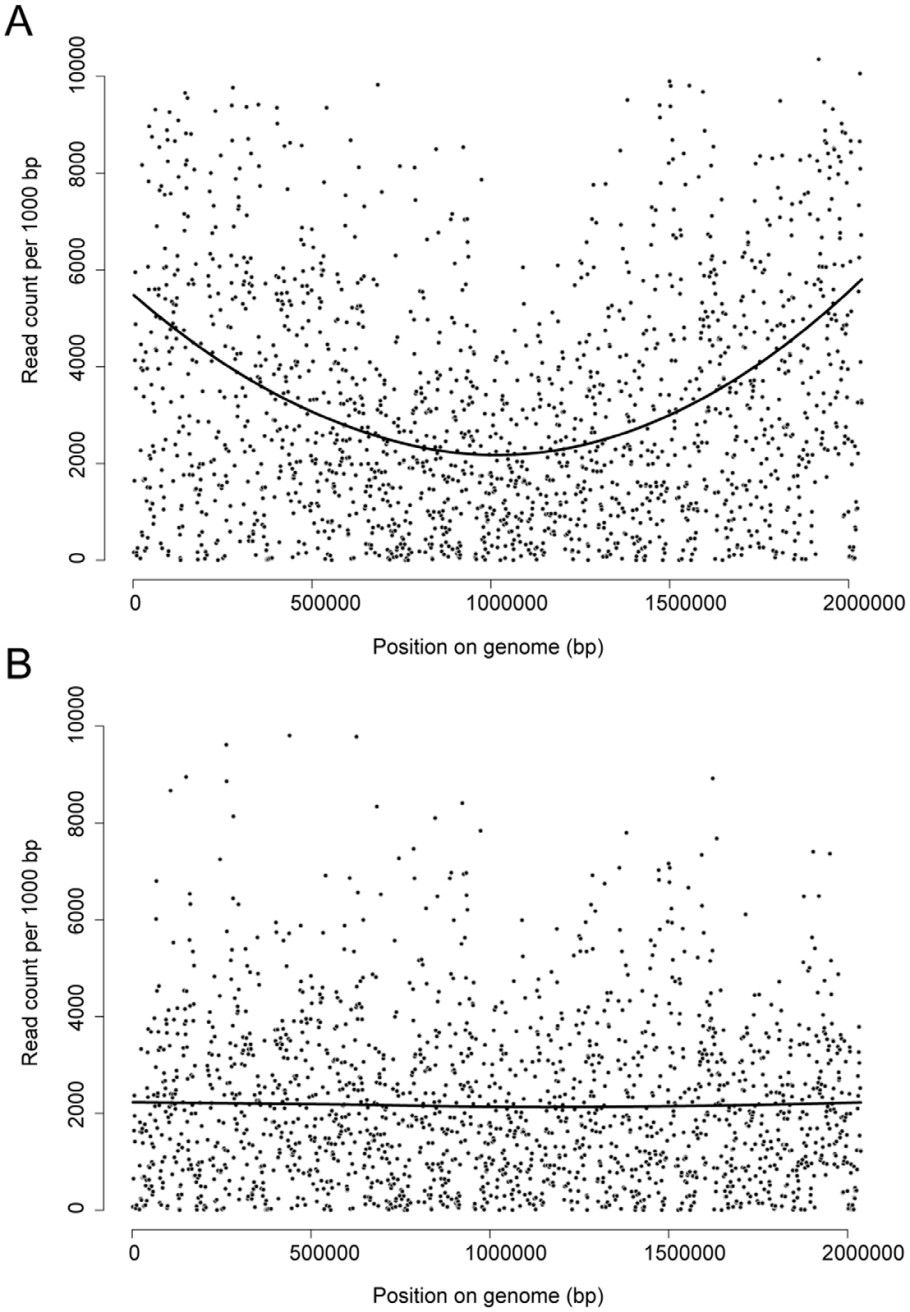
Read count as a function of genomic position per 1 kb. Read count of a single Tn-seq experiment of *S. pneumoniae* R6 gene essentiality as a function of the genomic position before (A) and after (B) genomic location correction using Loess. Each dot represents 1 kb of sequence. Regression on the data was performed using Loess as implemented in the loess R package and plotted on the graph as a black line.

Conceivably, because of genomic replication during growth and the resulting increase in available DNA close to the origin of replication (ORI), read counts increase as they are closer to the ORI, especially if multiple replication forks are present [32]. Additionally, more genomic DNA close to the ORI is available for transposon mutagenesis, resulting in a higher number of transposon insertions closer to the ORI. These two factors produce a substantial bias, showing up as typical V-shape in the read counts per gene relative to the genomic location, with average read counts at the ORI being at least 3 times higher than those near the terminus of replication (Fig. 2A). Gallagher *et al.* [10] noticed a similar bias in their study when *P. aeruginosa* PAO1 was exposed to tobramycin. They corrected for this bias by calculating the local read density within a 100-kb window and normalizing the number of reads at that position relative to the average local read density for that window.

Because the local read density window needs to be optimized for genome size and mutant library size, we opted instead to correct for this bias by using locally weighted scatterplot smoothing (LOESS) on read counts per insertion site and per gene as function of genomic position. By applying the ratio between the LOESS curve and a straight line of the average signal to the read counts, the read count bias per insertion site and per gene (Fig. 2B) was alleviated without the requirement of parameter optimization. Using this approach, a better separation of non-essential and essential genes was observed (Table 1, Fig. 1A and B) for both *S. pneumoniae* R6 and *P. aeruginosa* PAO1, reflected in decreased p-values and a perfect AUC for *S. pneumoniae* R6 and a higher AUC than the prediction by Gallagher *et al.* for *P. aeruginosa* PAO1.

### ESSENTIALS can be Used for different Transposon Mutagenesis Sequencing Methods

In order to validate ESSENTIALS as a tool for general use we have analyzed several transposon sequencing literature datasets obtained from SRA at EBI [33] that were based on different transposon insertion sequencing technologies. We compared the results of ESSENTIALS on gene essentiality with the results presented in the respective studies using either the optimal fold change under-representation cut-off predicted by ESSENTIALS or the same fold change cut-off as applied in the study [34]–[36] and a FDR corrected p-value <0.05 cut-off. Because the results obtained with the reference sets (Table 1) produced, in the case of *S. pneumoniae* R6, a 100% correct prediction, and for *P. aeruginosa* PAO1, a better prediction than presented by the original authors [10] we are confident in suggesting ESSENTIALS performs equally well or better in detecting (conditionally) essential genes using the optimal settings (Table 1, in bold). Although a direct comparison of the number of false positives and false negatives cannot be made because of the arbitrary fold change and P-value cut-offs or different analysis algorithms used in the studies describing these datasets, ESSENTIALS allowed determination of the vast majority of the previously reported conditionally essential genes (Table 2). Additionally, the flexibility of ESSENTIALS in selecting how to process the various read file formats allows the analysis of all known transposon insertion sequencing analysis methods.

**Table 2:** The use of ESSENTIALS on data generated by various transposon sequencing techniques.

Strain	Condition	Number of essential genes/Log2 FC cut-off$					Method	Ref
		Literature		ESSENTIALS		Overlap		
		N	FC	N	FC	N		
*S. pneumoniae* TIGR4	essential	396	NA	423	−4.1	357	Tn-seq	[43]
*S. typhi* Ty2	essential	356	NA	335	−3.71	323	TraDIS	[44]
	bile salt	169	−1.40*	229	−1.40#	161	TraDIS	
*H. influenzae*	essential	358	−4.32	383	−3.2	344	HITS	[45]
Rd	*in vivo*	141	−1.79	130	−1.79#	100	HITS	

$ Optimal fold change (FC) underrepresentation cut-offs detected by ESSENTIALS; N: number; NA: Not available, a different method was used to determine gene essentiality in these studies.

# A minimum normalized average read count of 50 reads per gene was required; FC cut-offs were the same as used in the literature reference to facilitate comparison.

* Although the authors state in their methods that a −2 log2 fold change and a p<1*10-5, adj. p<2.5E-4 cut-off was used, only the p-value cut-offs were applied, resulting in a -1.4 fold change cut-off (personal communication Julian Parkhill).

## Methods

ESSENTIALS is implemented in Perl v5.8.8 and R v2.14.1. Its web interface is generated by the FG-web framework (van Hijum *et al*., https://trac.nbic.nl/fgweb/). The ESSENTIALS algorithm downloads and preprocesses sequencing read files, matches sequenced transposon flanking reads to insertion sites on the genome and then performs various processing steps and statistics on the resulting transposon insertion count data. Via email the progress of the run is reported.

There are three major sections in the web-interface: (i) configuration file upload and genome selection; (ii) parameter settings; and (iii) displaying the results. The web tool works with major web browsers such as Internet Explorer, Firefox, Safari and Opera. It can be tuned to the needs of a researcher by modifying several parameters controlling the alignments, normalization, statistical tests and visualization.

### Input Data

**Figure 3:**
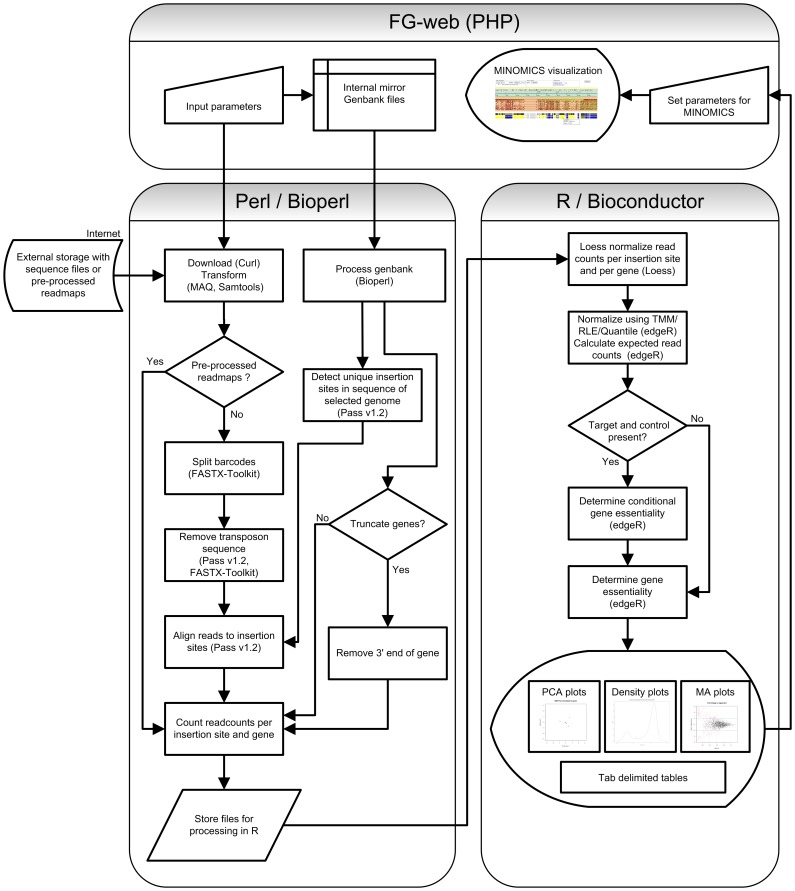
Simplified flowchart of the ESSENTIALS procedure. Links to sequence reads files are uploaded and parameters are optionally changed via the FG-web interface that works on most web-browsers. It allows users to perform multiple runs at the same time through session management. As processes are queued, users can start multiple analyses at the same time, and check the progress via web-pages that can be bookmarked.

A simplified flow chart of the procedure followed by ESSENTIALS is shown in Figure 3. The genome of the organism that was used to create the knockout library for the Tn-seq can be selected either by (i) selecting from the available daily updated Genbank sequences or, (ii) uploading a Genbank file. A tab-delimited configuration file should be uploaded or can be generated using the ESSENTIALS web-interface. The configuration file should contain the following information: (i) a hyper link to the sequence reads, (ii) the barcode sequence, if used, (iii) transposon sequence (if used), (iv) condition, (v) knockout library, (vi) sequence file format (FASTA, FASTQ, EXPORT, SCARF, CSFASTA, BAM, SAM or a custom tab delimited file) and (vii) used compression (none, zip, gzip, bzip2). After supplying ESSENTIALS with the sample descriptions and additional information the pre- and post-processing steps of the algorithm can be set by the user and the analysis can be started.

### Algorithm

The ESSENTIALS algorithm matches sequenced transposon flanking reads to insertion sites on the genome and then performs statistics on the resulting counts data. A stepwise explanation of the procedure follows below. When a BAM, SAM or a tab delimited file containing the read counts per insertion site is uploaded, the filtering and aligning procedures are skipped and the count data per gene is generated from the user supplied mappings.

**Step 1.** All (unique) putative insertion sites (either random or *mariner* transposon TA insertion sites) are detected on the genome of interest and sequences adjacent to each insertion site are entered into a FASTA file.

**Step 2.** Sequence read files are downloaded, uncompressed and transformed to FASTA using a modified version of fq_all2std from MAQ (http://maq.sourceforge.net/). Read files are split on barcodes using a modified version of fastx_split from the fastx toolkit (http://hannonlab.cshl.edu/fastx_toolkit/) that uses Levenshtein distance instead of Hamming distance to allow fuzzy matching of barcodes of different lengths. When necessary sequence reads are filtered on the presence of transposon repeats using PASS v1.2 [37] or fastx_split for long (>12) or short transposon ends ( = <12), respectively from either the 5′ or 3′ end.

**Step 3.** The transposon-flanking genomic sequence reads are aligned to the insertion sites using PASS and count data is generated per unique insertion site and per gene. Reads that map on more than one place on the genome can be removed from the analysis. Reads that map in the 3′ end of a gene can also be filtered out to remove transposon insertions that do not cause loss of function. Genomic location insertion and read bias is corrected by LOESS regression on read counts per insertion site and per gene relative to genomic location.

**Step 4.** Count data is combined for analysis with EdgeR [38] and normalization is performed using either scaling, trimmed mean of M-values (TMM), Relative Log Expression (RLE) [20] or Quantiles [39].

**Step 5.** Read counts per gene or per insertion site of the control and target samples are tested for significant difference to determine conditional essentiality of genes or insertion sites. Obligate gene essentiality is determined by comparing the expected number of reads per gene (based on the number of insertion sites per gene, the mutant library size and the sequencing depth) and the measured number of reads per gene. Significantly underrepresented genes are considered (conditionally) essential. ESSENTIALS uses the negative binomial distribution statistical model in EdgeR, an exact test or a General Linearized Model likelihood ratio test and estimates the dispersion in the data with quantile-adjusted conditional maximum likelihood (qCML) or Cox-Reid profile-adjusted likelihood (CR). The qCML method is applied to experiments with a single mutant library and the CR method to experiments with multiple libraries. The log2 transformed ratio of target over control or measured over expected and signal of (conditionally) essential genes or insertion sites is then used to generate kernel density plots using a Gaussian model with stepwise increasing bandwidth and 2048 bins until a single (in the case of essential genes) or four (in the case of conditional essential genes) local minima are found. Local minima are detected by calculating the first derivative of the density and by locating the position where it traverses from values below to values above zero. This fold change value corresponds to a value closest to the minimum between the peaks of essential and non-essential genes and can be used as a cut-off to determine whether a gene is essential or not.

### Output of ESSENTIALS

Results of ESSENTIALS include: (i) Principal Component Analysis (PCA) plots, which can be used to explore the similarities between the target and control samples based on the number of reads per gene or per insertion site; (ii) Signal to log2-ratio (MA) plots which can be used to visually inspect the fold ratio of target over control or measured over expected and signal of (conditionally) essential genes or insertion sites; (iii) Density plots of the ratio of target over control or measured over expected and signal of (conditionally) essential genes or insertion sites with detected putative fold change cut-offs for selection of essential genes; (iv) tab delimited tables containing the raw counts, normalized counts, output from the statistical tests and genomic information and; (v) Links to visualize the results in MINOMICS [40], also implemented in the FG-web framework.

### Availability

The web-interface of ESSENTIALS, the output from the various analyses presented in this paper and an optional demo mode, analyzing a subset of the *S. pneumoniae* R6 dataset, can be accessed freely at http://bamics2.cmbi.ru.nl/websoftware/essentials/. Source code is available via http://trac.nbic.nl/essentials/.

### Conclusions

Transposon insertion sequencing analysis is becoming the default method of high-throughput fitness screening in prokaryotes. Emergence of next-generation sequencing based approaches such as transposon insertion sequencing analysis and high-throughput random RNAi interference screens in eukaryotic organisms [41], [42] will lead to similar data types. ESSENTIALS provides an easy to use and automated method to rapidly analyze these datasets. Prediction of gene essentiality by ESSENTIALS is comparable or possibly better than that reported by the original authors because ESSENTIALS applies data filtering, normalization and suitable statistical tests that are optimized to recover as many as possible essential genes. ESSENTIALS will greatly benefit researchers performing these studies saving both time and providing robust, yet sensitive detection of essential genes from transposon insertion sequencing analysis experiments.

## Supporting Information

Table S1 **Input data for ROC analysis and statistical tests for performance evaluation of** ***S. pneumoniae*** **R6 gene essentiality prediction.**
(XLSX)

Table S2 **Input data for ROC analysis and statistical tests for performance evaluation of** ***P. aeruginosa*** **PAO1 gene essentiality prediction.**
(XLSX)

Text S1 **Detailed Materials and Methods.**
(DOCX)
