# Supplementary material for: ESSENTIALS: Software for Rapid Analysis of High Throughput Transposon Insertion Sequencing Data
Source: PLoS One. 2012 Aug 10;7(8):e43012. doi: 10.1371/journal.pone.0043012 (PMC3416827; doi:10.1371/journal.pone.0043012)
Supplement: Text S1 — Detailed Materials and Methods. (DOCX) [file pone.0043012.s003.docx]

**Supplemental text S1**

**Bacterial strains and growth conditions.** The bacterial strains used in this study are listed in Table 1. *S. pneumoniae w*as routinely grown in Brain Heart Infusion (BHI) broth (Difco) with 200 U ml^-1^ bovine liver catalase (Sigma) or on Colombia agar (Oxoid) plates supplemented with 5% sheep blood (Biotrading) at 37°C and 5% CO_2_. *Escherichia coli* was grown on Luria Bertani (LB) agar plates or in LB broth in a shaking incubator at 37°C. *S. pneumoniae* and *E. coli* genetic transformation was performed as described previously [1]. When indicated, antibiotics were used at the following concentrations: spectinomycin, 150 µg ml^-1^ for *S. pneumoniae*; kanamycine 50 µg ml^-1^ for *E. coli*.

**Table 1. Bacterial strains and plasmid in this study**

| **Strains and plasmids** | **Relevant characteristics** | **Reference** |
| --- | --- | --- |
| Strains |  |  |
| *S. pneumoniae* R6 | Wild-type strain, unencapsulated | [2] |
| *E. coli* DH5a | Cloning strain | [3] |
|  |  |  |
| Plasmids |  |  |
| pR412 | Donor for Sp^r^ cassette, Amp^r^, Sp^r^ | [1] |
| pR412T7 | Donor for *marinerT7* transposon, Amp^r^, Sp^r^ | [4] |
| pGSF8 | Donor for *marinerT7*-MmeI transposon, Amp^r^, Sp^r^, Km^r^ | This study |

Sp^r^, spectinomycin resistant; Km^r^, kanamycin resistant; Amp^r^, ampicillin resistance

**DNA extraction, PCR amplification, and DNA quantification.** Chromosomal DNA was isolated from *S. pneumoniae* broth cultures with the Qiagen Genomic tip (Qiagen). Plasmids were isolated from *E. coli* broth cultures with a Qiaprep mini kit (Qiagen). For construction of Tn-seq plasmids and the *S. pneumoniae* directed-deletion mutants the proofreading *Pwo* DNA polymerase (Roche) was used. For the Tn-seq DNA probe synthesis, the Phusion DNA polymerase (Bioke) and HF buffer was used. For other PCR-based approaches, AmpliTaq DNA polymerase (Applied Biosystems) was applied. The primers (Biolegio, Nijmegen, Netherlands) that were used in this study are listed in Table 2. DNA concentrations of solutions were routinely measured with a Nanodrop (Thermo Fisher Scientific), but for the Tn-seq sequencing probes this was performed with a Qubit (Invitrogen).

**Table 2. Primers in this study**

| Primer Name | Nucleotide sequence (5’-3’) |
| --- | --- |
|  |  |
| *Plasmid construction* |  |
| PBGSF20 | P-ACAGGTTGGATGATAAGTCCCCGGTCT |
|  |  |
| *Tn-seq** |  |
| PBGSF23 | CAAGCAGAAGACGGCATACGAAGACCGGGGACTTATCATCCAACCTGT |
| PBGSF29 ATCACG | TTCCCTACACGACGCTCTTCCGATCTATCACGNN |
| PBGSF30 ATCACG | P-CGTGATAGATCGGAAGAGCGTCGTGTAGGGAAAGAGT-P |
| PBGSF29 CGATGT | TTCCCTACACGACGCTCTTCCGATCTCGATGTNN |
| PBGSF30 CGATGT | P-ACATCGAGATCGGAAGAGCGTCGTGTAGGGAAAGAGT-P |
| PBGSF29 TTAGGC | TTCCCTACACGACGCTCTTCCGATCTTTAGGCNN |
| PBGSF30 TTAGGC | P-GCCTAAAGATCGGAAGAGCGTCGTGTAGGGAAAGAGT-P |
| PBGSF29 TGACCA | TTCCCTACACGACGCTCTTCCGATCTTGACCANN |
| PBGSF30 TGACCA | P-TGGTCAAGATCGGAAGAGCGTCGTGTAGGGAAAGAGT-P |
| PBGSF31 | AATGATACGGCGACCACCGAGATCTACACTCTTTCCCTACACGACGCTCTTCCGATCT |
|  |  |

P, phosphorylated; *Tn-seq primers are PAGE purified;

**Plasmid construction.** The plasmids used in this study are listed in Table 1. To make the Genomic Array Footprinting (GAF) *marinerT7* transposon [4] suitable for transposon sequencing (Tn-seq) [5], the *marinerT7* transposon was PCR amplified from the pR412T7 plasmid with a single 5’-phosphorylated primer PBGSF20. PCR cycling conditions were as follows: 93°C for 4 min; 30 cycles of 93°C for 30s, 50°C for 30s, and 68°C for 2 min; and 68°C for 5 min. The PCR reaction introduced a MmeI endonuclease site in the inverted repeats of the *marinerT7* transposon. This *marinerT7-MmeI* transposon PCR product was cloned into the pCR2.1 vector of the TA cloning kit (Invitrogen) to obtain pGSF8. Correct integration of the MmeI site was confirmed by Sanger sequencing.

***S. pneumoniae* mutant library construction.** *S. pneumoniae* R6 *marinerT7-MmeI* transposon mutant libraries were generated essentially as described previously [4]. Briefly, 0.5 µg of *S. pneumoniae* genomic DNA was incubated for 4 hrs with purified HimarC9 transposase and 0.5 mg of pGSF8 plasmid. After repair of the resulting transposition products with T4 DNA polymerase and *E. coli* DNA ligase, 100 ng mutagenized DNA was used for transformation of 1 ml precompetent *S. pneumoniae* cells. For mutant library construction, the required number of colonies was scraped from the plates, pooled, grown to mid-log phase in 5% CO_2_-enriched BHI or GM17, and aliquots were stored with 15% glycerol at -80°C.

**Tn-seq analyisis of *S. pneumoniae* mutant libraries.** The Tn-seq technology was performed essentially as described previously [5] with minor modifications. Briefly, a 200-µl solution with 2 µg *S. pneumoniae* mutant library genomic DNA in NEBuffer 4 (New England Biolabs) with 5 mM S-adenosylmethionine was  digested with 10 U MmeI endonuclease (New England Biolabs) for 4 hrs at 37°C and dephosphorylated with 1 U calf intestine alkaline phosphatase (Invitrogen) for 30 min at 50°C. Next, the reaction was successively extracted with 200 µl phenol:chloroform:isoamyl alcohol (25:24:1), extracted with 200 µl chloroform:isoamyl alcohol (24:1), ethanol-precipitated, and the dried DNA pellet was dissolved in 20 µl H_2_O. Tn-seq adapters with a 6-bp barcode were prepared by combining 5 nmol of two matching PBGSF29‘barcode’ and PBGSF30‘barcode’ oligonucleotides in 1xTE and 50 mM NaCl in a total volume of 50 µl, a 10-min denaturation step at 95°C, and annealing step in which the reaction was slowly cooled to room temperature. A 20-µl solution with 200 pmol adapter was phosphorylated with T4 polynucleotide kinase (3' phosphatase minus) (New England Biolabs) in T4 ligase buffer (New England Biolabs) for 5 min at 37°C, and heat-inactivated for 10 min at 70°C. Ligation of 100 ng dephosphorylated MmeI restriction fragments with 2 pmol phosphorylated adapter was performed in the presence of T4 DNA ligase buffer with 2 U T4 DNA ligase (New England Biolabs) in a total volume of 20 µl for 1 hr at 16°C. Immediately after the ligation, Tn-seq DNA probes were generated by PCR with 2.5 µl ligation reaction as template, 20 pmol PBGSF23 and PBGSF31 primers, HF buffer, 0.2 mM dNTP mix, and 1 U Phusion DNA polymerase in a total volume of 50 µl. PCR cycling conditions were as follows: 72°C for 1 min, 98°C for 30s; 25 cycles of 98°C for 30s, 57°C for 30s, and 72°C for 10 s; and 72°C for 5 min. The resulting PCR product of ~130 bp was purified from the PCR reaction with the Minelute Reaction Cleanup Kit (Qiagen). After pooling of samples with up to 4 different 6-bp barcodes, typically 9 fmol Tn-seq DNA probes was loaded on a Genome Analyzer II (Illumina) for sequence analysis with the manufacturer’s protocol, using the Genomic DNA Sequencing Primer (Illumina), and 36 sequencing cycles.

**Data analysis.** For Tn-seq data analysis FASTA or FASTQ files were imported in ESSENTIALS (bamics2.cmbi.ru.nl/websoftware/essentials) after removal of the barcode and transposon sequences and processed as described in the main manuscript text. Read counts per gene or per insertion site of the control and target samples were tested for significant differences in EdgeR [6] by quantile-adjusted conditional maximum likelihood (qCML) method assuming common dispersion of variance. P-value were adjusted as proposed by Benjamini & Hochberg [7] to correct for multiple testing. Gene essentiality was determined by comparing the expected number of reads per gene (based on the number of insertion sites per gene, the mutant library size and the sequencing depth) and the measured number of reads per gene. Significantly underrepresented genes (P <0.05, Fold change cutoff as predicted by ESSENTIALS or as used in the literature references) were considered essential.

**Reference List**

1. Martin B, Garcia P, Castanie MP, Claverys JP (1995) The *recA* gene of *Streptococcus pneumoniae* is part of a competence-induced operon and controls lysogenic induction**.** Mol Microbiol 15:367-379.

2. Hoskins J, Alborn WE, Jr., Arnold J, Blaszczak LC, Burgett S et al. (2001) Genome of the bacterium *Streptococcus pneumoniae* strain R6**.** J Bacteriol 183:5709-5717.

3. Hanahan D (1983) Studies on transformation of *Escherichia coli* with plasmids**.** J Mol Biol 166:557-580.

4. Bijlsma JJ, Burghout P, Kloosterman TG, Bootsma HJ, De JA et al. (2007) Development of genomic array footprinting for identification of conditionally essential genes in *Streptococcus pneumoniae***.** Appl Environ Microbiol 73:1514-1524.

5. van Opijnen T, Bodi KL, Camilli A (2009) Tn-seq: high-throughput parallel sequencing for fitness and genetic interaction studies in microorganisms**.** Nat Methods 6:767-772.

6. Robinson MD, McCarthy DJ, Smyth GK (2010) edgeR: a Bioconductor package for differential expression analysis of digital gene expression data**.** Bioinformatics 26:139-140.

7. Benjamini Y, Hochberg Y (1995) Controlling the False Discovery Rate: A Practical and Powerful Approach to Multiple Testing**.** Journal of the Royal Statistical Society Series B (Methodological) 57:289-300.
